# A survey study of physical activity participation in different organisational forms among groups of immigrants and descendants in Denmark

**DOI:** 10.1186/s12889-025-21314-5

**Published:** 2025-01-28

**Authors:** Eva Berthelsen Schmidt, Karsten Elmose-Østerlund, Bjarne Ibsen

**Affiliations:** Centre for Sports, Health, and Civil Society, Research Unit for Active Living, Department of Sports Science and Clinical Biomechanics, University of Southern Denmark, Odense, Denmark

**Keywords:** Ethnicity, Sociodemographic characteristics, Socioeconomic characteristics, Sports participation, Sports clubs, Commercial centres, Self-organised activities, Integration policy

## Abstract

**Background:**

Several studies have found that immigrants and descendants are less physically active than the majority population, particularly within sports clubs. However, most studies do not provide breakdowns by specific ethnic groups or organisational forms. Therefore, our paper analyses the influence of ethnicity, immigrant status, and sociodemographic and -economic characteristics on the physical activity participation of immigrants and descendants in sports clubs, commercial centres and self-organised activities in Denmark.

**Methods:**

Data were collected through a survey study conducted in 2020, with responses from approximately 163,000 adults, of whom eight percent had an immigrant or descendant background. We conducted both a descriptive analysis of the dependent and independent variables and multiple logistic regression analyses, one including the entire sample (including citizens of Danish origin) and one including only citizens with an immigrant or descendant background.

**Results:**

Our analysis revealed that immigrants and descendants, particularly those of non-Western origin, were significantly less likely than ethnic Danes to be active within the three organisational forms, with the smallest differences observed regarding participation in commercial centres. The analysis also revealed that besides ethnicity, immigrant status and sociodemographic and -economic characteristics were relevant in explaining differences in physical activity participation.

**Conclusion:**

Our study makes a significant contribution to the literature on physical activity participation among immigrants and descendants from various backgrounds. By utilising a robust sample size and employing statistical analysis, we offer novel insights into participation patterns that have traditionally been explored more qualitatively. Besides the need to study physical activity participation in different organisational forms among different ethnic backgrounds, we also highlight the importance of considering immigrant status and sociodemographic and -economic characteristics in understanding participation behaviours. These findings imply the importance of adopting a holistic and nuanced approach to promoting physical activity participation among immigrant and descendant populations, considering the multifaceted nature of the barriers and facilitators influencing engagement within different organisational settings.

## Background

Due to the influx of immigrants to most European countries in recent decades, integration policies have become a highly debated topic in Europe. Political debates regarding if and how to integrate immigrants and descendants into the hosting societies are present in many countries [e.g. [1–4]]. From a sociological perspective, integration of immigrants and descendants happens when social relations and networks are established within the host country [5, 6]. Considering this, civil society organisations are often highlighted as important for bringing people of different ethnic backgrounds together [7], and the participation is seen as a step towards integration [8]. Policy documents often target physical activity participation, most often in sports clubs, as an important vehicle for integration of immigrants and descendants [1, 9]. Several scholars have described sports clubs as ideal settings for encounters between people with cultural differences that reduce prejudices through interethnic contact [3, 10] and promote social cohesion, network formation, civic engagement and social capital, which is conducive to generalised trust, among other things, due to the regular participation and social aspect of sports practice, which is more prominent in sports clubs compared to other organisational forms [11–15]. However, it is also recognized that social closure practices and conflicts can occur in settings for organised physical activity participation, including sports clubs [16, 17].

Considering the integrative potential and other welfare and health benefits of physical activity participation, it can be viewed as problematic when several studies find immigrants and descendants to be less physically active than the majority population [4, 18, 19]. However, most studies do not provide breakdowns by specific ethnic groups. To understand what can explain differences in the physical activity pattern of immigrants and descendants, the heterogeneity of this group must be considered. The same can be said about the heterogeneity of physical activity participation, particularly in relation to the organisational form in which it is practiced. Sports clubs are one relevant organisational form, due to many participants and extensive attention in previous research and in political debates. But according to Hallmann, Feiler & Breuer [20] and Skinner et al. [21] it is important to consider different offers and inter-organisational linkages, as ‘a one-size-fits-all-approach’ will not meet all community and individual needs.

To mitigate the described limitations, it is the purpose of this article to provide a detailed picture of the role that ethnicity, immigration status and sociodemographic and -economic characteristics play for the physical activity participation of immigrants and descendants in sports clubs, commercial centres and self-organised activities in Denmark. This will generate differentiated knowledge about the traits that are conducive to participation in different organisational forms of physical activity, which can inspire policies that seek to increase the physical activity participation of immigrants and descendants.

### Immigrants, descendants and policy in Denmark

In Denmark, immigrants are defined as being born abroad and neither of the parents are both Danish citizens and born within the country. The category covers a diverse group, including economic migrants as well as refugees and asylum seekers, and are often described as separate categories, yet the various types of migrants share the transnational experience. Descendants are born in Denmark and neither of the parents are both Danish citizens and born within the country [22]. Compared to other European countries, immigrants and descendants make up a relatively low percentage of the population in Denmark [23]. Nevertheless, Denmark has seen a growth in the number of immigrants and descendants from 3 percent of the population in 1980 to 15 percent in 2023, which is equivalent to 910,898 people. Immigrants and descendants with a Western background make up 36 percent of the immigrants and descendants in Denmark, while immigrants and descendants with a non-Western background make up 64 percent, and half thereof – 32 percent – come from the so-called MENAPT countries (Middle East and North African countries and Turkey). Due to an increased influx of immigrants and descendants from MENAPT countries in recent years, the Danish integration policy places a significant emphasis on the need for integration of immigrants and descendants with origin in MENAPT countries and the challenges connected to this [24].

Over the last twenty years, changing governments in Denmark have pursued a very strict refugee and immigration policy, and there has been relatively strong support for this in the population [25]. The attitude towards migrants and refugees in Denmark is ambivalent [26, 27]. The basic attitude of the population is that not too many refugees and immigrants should come to Denmark, but those who come should be treated properly, but not get ‘special treatment’. The goal for the integration of migrants in Denmark is a) that newly arrived foreigners are ensured the opportunity to participate on an equal footing with other citizens in society's political, economic, working, social, religious and cultural life, b) that newly arrived foreigners become self-sufficient as soon as possible through employment, and c) that the individual foreigner gains an understanding of the fundamental values and norms of the Danish society. In that context, it is further recognized within the Danish integration policy that especially participation in sports clubs, can contribute to the integration of immigrants and descendants into society, and initiatives to promote integration through sport are almost exclusively targeted at sports clubs [9].

This highlighting of sports clubs align closely with the Danish sports and exercise policy, which have a focus on ‘Sport for all’, but first and foremost have the goal to promote and support physical activity organised in sports clubs. This happens partly through state support for the sports organisations, partly through municipal support for sports clubs and especially the facilities that sports clubs use. The aim of this policy is partly to promote physical activity participation, partly to strengthen citizenship and democratic formation (purpose of the municipal support for sports) [28]. The public sector does not provide financial support for commercially organised physical activity but has recently established a number of outdoor facilities that can be used by citizens for self-organised physical activities.

### Physical activity and organisational forms in Denmark

Denmark is among the countries in Europe with the overall highest physical activity participation rates [29]. The most popular organisational forms for physical activity in Denmark are sports clubs, commercial centres and self-organised activities. Sports clubs are associations characterized by voluntary membership, unpaid labour, democratic decision-making structures, and a not-for-profit orientation [30]. Commercial centres (e.g. fitness centres) are characterized by being privately owned, having a for-profit orientation, and the participants having no formal influence on the organisation. Self-organised activities include physical activity outside an organisational setting and includes participation on your own or in informal groups [31, 32]. While the proportion of people being physically active in a sports club has been slightly decreasing over the past decade, participation in commercial and self-organised physical activity has increased [33]. Like other countries, Danish studies also find immigrants and descendants to be underrepresented regarding physical activity participation across the different organisational forms [34, 35].

### Conceptual framework

This article seek to answer four research questions regarding immigrants’ and descendants’ physical activity participation in sports clubs, commercial centres, and self-organised activities. In the following, we present the research questions and elaborate on the theoretical and empirical justifications that led to these.

#### Ethnicity

One of the possible explanations for the lower physical activity participation rates of immigrants and descendants is that the extent of participation in physical activity, and especially the way in which it is practiced, is culturally determined: that is, influenced by norms, values, traditions, and ways of life that a person has grown up with [36, 37]*.* Among other things, the value that physical activity is given, the awareness of the health benefits, gendered attitudes toward appropriate behaviour, conflicts with religious beliefs and the like have been described as factors that inhibit some immigrants and descendants from being physically active in certain ways and contexts [35, 38–41]. However, studies also argue that ethnic background seem to play a greater role for preferences regarding the type of physical activity and organisational form rather than the overall physical activity level [42].

A broad range of characteristics is used when conceptualising the term ‘ethnicity’, often incorporating multiple criteria, such as language, physical similarities, styles of clothing, and religion. Other common elements in the existing definitions are a reference to shared heritage, roots or ancestry [43]. This reflects a distinction between ethnicity as a more subjective identification, aligning with the definitions described by Weber [44] in which ethnic groups are defined by “*a subjective belief in their common descent”*. This is opposed by a more objectively based identification, which is seen in the work of Bhopal [45] and Aspinall [46], who both argues that shared origin, common ancestry, or place of origin are central components of ethnicity. Each approach provides different classifications. Due to Danish registration standards classifying people in relation to either their citizenship, country of birth or country of origin, we follow the more objectively based understanding of ethnicity, which also aligns with recent trends within the field of health and physical activity research [e.g., [43, 47]]. Therefore, although ethnicity could be explored through more individualised variables, we use the country of origin as a proxy, leading us to the following research question (RQ):

*RQ1: How does country of origin influence immigrants' and descendants’ physical activity participation in different organisational forms?*

#### Immigrant status

Because immigrants are born outside of the host country, while descendants are born in the host country, it is likely that immigrants to a higher extent than descendants experience barriers related to participating in physical activity. This could be barriers such as a lack of understanding of language, a lack of social networks, experiences with discrimination, being unfamiliar with the ways certain activities are practiced, and insufficient knowledge of available opportunities [48]. However, it can be assumed that such barriers diminish as immigrants become more accustomed with norms and habits in the host country, and that physical activity participation over time will approach the movement habits in the majority population [40]. A process which is often described by the term acculturation, which can be defined as the merging of cultures because of prolonged contact between individuals and groups of different cultural backgrounds [49].

Even though acculturation should ideally be measured by culturally specific measures such as identity, attitudes, and language, most studies rely on proxies for acculturation such as duration of residence in the country or age upon arrival in the host country [50, 51]. Because these are not direct measures of acculturation, we do not claim to measure acculturation, but rather aspects found to be relevant regarding immigration status. Two reviews of the research into immigrants’ physical activity participation indicate that the duration of the immigrant’s residence and citizenship in the host country are associated with higher rates of participation in physical activity [49, 51]. Participation has also in the Netherlands been found to be influenced by how old the immigrant was upon the time of arrival in the host country, where those arriving at younger ages participate in recreational activities at higher rates than those who arrive when they are older [38].

Another relevant aspect of immigration status is the settlement of immigrants and descendants in the host country. In residential areas with relatively many immigrants and descendants, it is easier for immigrants and descendants to establish social networks with people from the same background and to maintain their cultural identity, which may limit their integration into the broader society [52]. But over time, many immigrants and descendants move to other residential areas with relatively fewer immigrants and descendants. A study by Alesina and Ferrara [53] found that after controlling for individual characteristics, immigrants’ and descendants’ participation in recreational activities are significantly lower in ethnically fragmented communities than in communities with a more homogeneous population composition predominantly comprised of the majority population. These findings led us to the formulation of two research questions (RQs), one regarding only immigrants including immigrant specific variables, and one regarding both immigrants and descendants:

*RQ2: How does the number of years lived within the host country and the age on arrival in the host country influence immigrants' physical activity participation in different organisational forms?*

*RQ3: How does the proportion within the residential area who have an immigrant background influence immigrants' and descendants' physical activity participation in different organisational forms?*

#### Sociodemographic and -economic characteristics

Socioeconomic status (SES) is part of the third possible explanation for the lower physical activity participation among immigrants and descendants, compared to the majority population within the host country. SES, which is primarily inspired by Max Weber’s conceptualization of inequality [44], is defined as a measure of one’s combined economic and social status and usually includes three measures: education, occupation, and income. Extensive research has demonstrated a correlation between SES and life circumstances and habits in different areas, including health and physical activity behavior [54, 55] This also applies to different ethnic groups [34, 38, 51, 53]. The primary explanations for this correlation are that SES partly affects the opportunities to live a healthy life and be physically active, and partly influences norms and behaviour due to the different socialization that takes place during childhood and youth.

However, demographic factors – age, gender, family composition and level of urbanisation – also influence physical activity participation. Studies from Germany and Denmark show that while young men with an immigrant background are as active in sports clubs as young men of the majority population, young women with an immigrant background are much less active in sports clubs than young women of the majority population [56, 57]. A Danish study also found that participation in sports clubs decreased with age among immigrants and descendants, which was not the case in the majority population [58]. Regarding family composition and the level of urbanisation, a study of ethnic minorities in the Netherlands found that being married, having children and living in highly urbanized areas has a negative impact on the level of participation in recreational activities [38]. This led us to the following research question (RQ):

*RQ4: How does educational level, employment, income, age, gender, family composition and level of urbanisation influence immigrants’ and descendants’ physical activity participation in different organisational forms?*

## Methods

### Research design, data collection, and sample

Data are drawn from a survey examining the physical activity habits among the adult population in Denmark as part of a research project named ‘Moving Denmark’. The data were collected from mid-October to the end of November 2020. In the period of data collection, many countries had, due to the COVID-pandemic, restrictions concerning physical activity in public and non-public institutions and areas. Because the respondents were asked to think about their physical activity participation the last year, which is beyond the COVID-pandemic period, and because the conditions at the time of the survey were relatively normal for sports participation and physical activity in Denmark due to low infection numbers [59], we argue that the answers are most likely to be fairly similar to answers from a period without COVID. This is substantiated by the fact that studies in Denmark have shown that the citizens’ level of physical activity at the time of our survey was relatively little affected by COVID [60].

Following the data collection, the survey data were, with help from a personal identification number on all the respondents, combined with information from Statistics Denmark about each respondent, mainly regarding immigrant status and sociodemographic and -economic characteristics. The study and its data management procedures were approved by the University of Southern Denmark’s Research & Innovation Organization (approval number 10.680), and all research was carried out in accordance with the Helsinki Declarations. More about ethics approval and consent to participate can be read under the declarations.

The survey was distributed using digital mail (‘e-Boks’), which is a tool developed for safe communication between citizens and public authorities in Denmark. A total of 404,000 citizens in Denmark aged 15 years and older received the questionnaire, and approximately 163,000 citizens replied, corresponding to a response rate of 40 percent. The sample included 52,000 citizens with an ethnic origin other than Danish, of which about 14,000 answered the questionnaire, corresponding to a response rate of 27 percent for this group. These 14,000 responses constitute the data basis for the subsequent analyses of immigrants’ and descendants’ physical activity participation.

### Measurements

To identify in which organisational forms the respondents were physical active, the questionnaire contained questions regarding which activities the respondents engaged in during their leisure time. Physical activity during leisure time is often connected to sports and exercise activities but does in our questionnaire also includes activities such as walking, cycling on an ‘everyday bike’ and various outdoor activities such as hunting and fishing. For each activity the respondent practiced at least once a week, the respondent was asked about the organisational form in which the activity was practiced. Based on participation rates, we limit our focus to the three largest organisational forms: sports clubs, commercial centres, and self-organised activities. In sports clubs, the most frequently practised activities are strength training, football, gymnastics and badminton, in commercial centres it is strength training, fitness or cardio training and yoga, and as self-organised activities we find walking in moderate pace, walking with a dog, cycling on an ‘everyday bike’ and running to be the most popular activities. These variables on organisational form constitute the dependent variables and are used as dichotomous variables, where those who are active were defined as the respondents who reported to practice at least one activity at least weekly in each organisational form.

Three blocks of independent variables, related to the conceptual framework, are included in the statistical analysis: 1) Ethnicity, 2) Immigrant status, and 3) Sociodemographic and -economic characteristics. Regarding ethnicity, we differentiate according to country of origin in seven groups: 1) citizens of Danish origin, 2) descendants of Western origin, 3) immigrants of Western origin, 4) descendants with origin in MENAPT countries (Middle East and North African countries and Turkey), 5) immigrants with origin in MENAPT countries, 6) descendants of other non-Western origin, and 7) immigrants of other non-Western origin. This division into seven origin groups, are exclusively based on prior studies and the policy focus relating to integration of immigrants with origin in MENAPT countries in Denmark [24].

We operationalize immigrant status as: 1) the number of years the immigrant has been living in Denmark, and 2) the immigrant’s age on arrival in Denmark. These two variables can only be included in analyses that are conducted solely on immigrants. We also include the demography of the local area in which the respondents are living, which is divided into two variables: 3) the proportion of immigrants and descendants with origin in Western countries, and 4) the proportion of immigrants and descendants with origin in MENAPT and other non-Western countries. These two variables are based on data for each postal district in Denmark (defined by a postal code), provided by the Ministry of Immigration and Integration.

Finally, we examine sociodemographic and -economic characteristics with seven variables: 1) gender, 2) age, 3) educational level, 4) employment status, 5) income, 6) family composition, and 7) degree of urbanisation. Degree of urbanisation is divided into four categories, which was pragmatically formed based on a relatively equal number of respondents in each group. Descriptive statistics for the variables are shown in Table 1.

**Table 1:** Descriptive statistics, including mean values and standard deviations or frequency for all included variables

	**M (SD)**	**%**	**N**
**Organisational forms**			
Physically active in sport clubs		28.3%	157,414
Physically active in commercial centres		19.5%	157,414
Physically active in self-organised activities		74.4%	157,414
**Ethnicity**			
Citizens of Danish origin		91%	157,137
Descendants of Western origin		0.3%	157,137
Immigrants of Western origin		3.8%	157,137
Descendants with origin in MENAPT countries		0.8%	157,137
Immigrants with origin in MENAPT countries		1.5%	157,137
Descendants of other non-Western origin		0.4%	157,137
Immigrants of other non-Western origin		2.1%	157,137
**Immigrant status**			
Number of years lived in Denmark (immigrants only)	15.45 (13.48)		12,390
Age at arrival to Denmark (immigrants only)	26.93 (11.31)		12,390
Proportion of immigrants and descendants with origin in Western countries within the area	4.99 (2.56)		154,280
Proportion of immigrants and descendants with origin in MENAPT and other non-Western countries within the area	8.74 (6.40)		154,280
**Sociodemographic and -economic characteristics**			
Gender (male)		49.2%	157,414
Age (from 15 years)	48.16 (19.40)		157,414
**Education**			
Higher education		39.9%	156,178
High school and vocational education		40.0%	156,178
Primary school		20.1%	156,178
**Employment status**			
Studying		12.8%	157,379
Employed		55.2%	157,379
Social benefit recipients (retired or on a leave)		25.3%	157,379
Unemployed and cash benefit recipients		4.7%	157,379
Others		2.0%	157,379
**Income**			
Low income (€27,000 or less)		22.3%	157.414
Medium income (€27,000 – 42,000)		35.2%	157.414
High income (more than €42,000)		42.5%	157.414
**Household composition**			
Living without a partner without children living at home		25.1%	156,999
Living without a partner with children living at home		5.8%	156,999
Living with a partner without children living at home		37.6%	156,999
Living with a partner with children living at home		31.2%	156,999
**Urbanisation level**			
0–85 inhabitants/km^2^		25.9%	157,414
86–150 inhabitants/km^2^		22.8%	157,414
151–700 inhabitants/km^2^		21.0%	157,414
More than 700 inhabitants/km^2^		30.4%	157,414

Note: The total sample size (*N*) is 157,414, representing the number of respondents who answered questions about their physical activity during leisure time. However, some variation exists across the independent variables due to coding differences and missing information. The explanations for missing values are as follows: Ethnicity: Individuals with no registered country of origin are coded as missing. Number of years in Denmark and age at arrival: Only immigrants are included. Demography within the areas: Not registered is coded as missing. Education: Not registered and preschool education are coded as missing. Employment: Not registered is coded as missing. Household composition: Not registered is coded as missing

### Data analysis

Because our sample was disproportionally selected from a stratified population according to municipality size, we weighted the data accordingly. Furthermore, we found men and young people to be underrepresented among the respondents, which also led us to weight the data according to these differences.

Besides conducting a descriptive analysis of the dependent and independent variables (Table 1), we conducted multiple logistic regression analyses. The first regression analysis (Table 2) is conducted using the entire sample to enable comparisons between citizens of Danish origin and citizens with a background as immigrant or descendant. The second regression analysis (Table 3) is conducted only on citizens with a background as immigrant or descendant to examine similarities and differences in the effects from the independent variables on physical activity participation in comparison with Table 2. Further, Table 3 includes a regression analysis on immigrants only that enables the inclusion of the variables describing the number of years the immigrants have been living in Denmark and the immigrants’ age on arrival in Denmark within the category ‘immigrant status’.

**Table 2:** Logistic regression with participation in sport clubs, commercial centres, or in self-organised activities as dependent variables

	**Physically active in sport clubs**	**Physically active in commercial centres**	**Physically active in self-organised activities**
***Ethnicity (ref. citizens with Danish origin)***			
Descendants of Western origin	0.644***	1.135	1.0253
Immigrants of Western origin	0.730***	0.957	0.887***
Descendants with origin in MENAPT countries	0.679***	1.069	0.508***
Immigrants with origin in MENAPT countries	0.526***	0.921	0.428***
Descendants of other non-Western origin	0.687***	1.060	0.668***
Immigrants of other non-Western origin	0.532***	0.688***	0.670***
***Immigrant status***			
Proportion of immigrants and descendants with origin in Western countries within the area	0.979***	1.010***	1.012***
Proportion of immigrants and descendants with origin in MENAPT and other non-Western countries within the area	0.994***	1.001	,993***
***Sociodemographic and -economic characteristics***			
Gender (female)	0.888***	1.519***	1.322***
Age (from 15 years)	1.000	0.981***	0.999
Education *(ref. Higher education)*			
High school and vocational education	0.879***	0.825***	0.610***
Primary school	0.765**	0.634***	0.414***
Employment status *(ref. Studying)*			
Employed	0.618***	0.731***	0.670***
Social benefit recipients (retired or on a leave)	0.812***	0.787***	0.610***
Unemployed and cash benefit recipients	0.497***	0.755***	0.654***
Others	0.543***	0.778***	0.874**
Income (*ref. Low income—€27,000 or less*)			
Medium income (€27,000 – 42,000)	1.329***	1.187***	1.113***
High income (more than €42,000)	1.612***	1.495***	1.422***
Household composition *(ref. Living with a partner with children living at home)*			
Living without a partner without children living at home	0.791***	1.357***	1.006
Living without a partner with children living at home	0.856***	1.209**	0.970
Living with a partner without children living at home	0.871***	1.336***	1.133***
Urbanisation level *(ref. 0–85 inhabitants/km*^*2*^*)*			
86–150 inhabitants/km^2^	0.897***	1.135***	1.013
151–700 inhabitants/km^2^	0.871***	1.319***	1.031
More than 700 inhabitants/km^2^	0.850***	1.509***	1.083***
**Adjusted R2**	0.032	0.063	0.057
**N**	152,708	152,708	152,710

^***^p < 0.001, **p < 0.01, *p < 0.05; Odds ratios are presented

Note: All origin groups are included

**Table 3:** Logistic regression with participation in sport clubs, commercial centres or in self-organised activities as dependent variables

	**Physically active in sports clubs**		**Physically active in commercial centres**		**Physically active in self-organised activities**	
	Immigrants and descendants	Immigrants only	Immigrants and descendants	Immigrants only	Immigrants and descendants	Immigrants only
***Ethnicity (ref. immigrants of Western origin)***						
Descendants of western origin	0.835		1.134		1.1094	
Descendants with origin in MENAPT countries	0.865		1.009		0.530***	
Immigrants with origin in MENAPT countries	0.634***	0.622***	0.894	0.913	0.450***	0.442***
Descendants of other non-Western origin	0.882		0.973		0.715**	
Immigrants of other non-Western origin	0.713***	0.728***	0.708***	0.713***	0.764***	0.763***
***Immigrant status***						
Number of years in Denmark (immigrants only)^a^	-	1.003*	-	0.974***	-	0.999
Age on arrival to Denmark (immigrants only)^a^	-	0.989***	-	0.972***	-	0.998
Proportion of immigrants and descendants with origin in Western countries within the area	0.980*	0.983	0.995	0.996	1.015	1.011
Proportion of immigrants and descendants with origin in MENAPT and other non-Western countries within the area	0.987***	0.988**	0.997	0.996	0.994**	0.996
***Sociodemographic and -economic characteristics***						
Gender (female)	0.692***	0.756***	1.189***	1.328***	1.071	1.076
Age (from 15 years)^b^	0.996	-	0.972***	-	0.997	-
Education *(ref. Higher education)*						
High school and vocational education	0.796***	0.756***	0.941	0.917	0.661***	0.641***
Primary school	1.035	0.901	0.845*	0.818*	0.483***	0.484***
Employment status *(ref. Studying)*						
Employed	0.786**	0.931	0.945	1.002	0.687***	0.649***
Social benefit recipients (retired or on a leave)	1.096	1.132	0.972	1.025	0.693***	0.622***
Unemployed and cash benefit recipients	0.862	0.978	1.000	1.012	0.744**	0.670***
Others	0.713**	0.855	0.894	1.005	0.805*	0.775*
Income (*ref. Low income—€27,000 or less*)						
Medium income (€27,000 – 42,000)	1.110	1.033	1.183*	1.192*	1.005	0.996
High income (more than €42,000)	1.343***	1.216*	1.556***	1.576***	1.296***	1.271***
Household composition *(ref. Living with a partner with children living at home)*						
Living without a partner without children living at home	0.967	1.049	1.592***	1.804***	1.096	1.153*
Living without a partner with children living at home	0.852	0.778*	1.365**	1.646***	0.943	0.984
Living with a partner without children living at home	0.972	1.009	1.473***	1.547***	1.187**	1.235***
Urbanisation level *(ref. 0–85 inhabitants/km*^*2*^*)*						
86–150 inhabitants/km^2^	0.801**	0.778**	0.988	1.011	1.009	0.956
151–700 inhabitants/km^2^	0.861	0.837*	1.161*	1.169*	1.096	1.065
More than 700 inhabitants/km^2^	0.978	0.994	1.316***	1.378***	1.127	1.174*
**Adjusted R2**	0.030	0.029	0.064	0.068	0.084	0,0907
**N**	13,012	10,810	13,012	10,809	13,012	10,810

^***^p < 0.001, **p < 0.01, *p < 0.05; Odds ratios are presented

^a^These variables are only included in the model analysing only immigrants, as the variables are immigrant specific

^b^Age is not included in the model analysing only immigrants, as it conflicts with the variable ‘Age on arrival to Denmark’. Thus, the variable ‘Age on arrival to Denmark’ consist of two variables: 1) the date of arrival to Denmark and 2) the respondent’s age, both provided by Statistics Denmark. By having these two variables, we could create the variable by subtracting the respondents age with the year of the questionnaire distribution subtracted with the year of arrival (age – (2020 – year of arrival)

Note: Does not include citizens of Danish origin. The Full model includes both immigrants and descendants, while second model includes only immigrants

For both tables we see adjusted R^2^ values across all organisational forms between 0.03 to 0.09. These values suggest that while the models identify some significant factors influencing participation, much of the variability remains unexplained. Because the purpose of our study was to examine the role of ethnicity, immigrant status and sociodemographic and -economic variables in relation to physical activity participation in different organisational context, we did not search for predictors beyond the selected scope, even though this might have led to regression models with higher adjusted R^2^ values. Further, our models showed no violation of the assumptions of multiple logistic regression analysis.

## Results

We present the results from the logistic regression analyses reported in Tables 2 and 3 according to the three blocks of independent variables described in our conceptual framework.

### Ethnicity

Table 2 shows that all groups of immigrants and descendants are less likely to participate in sports clubs and self-organised activities than citizens of Danish origin. An exception is descendants of Western origin, where no significant differences compared to citizens of Danish origin are found. In sports clubs, there are particularly large differences between citizens of Danish origin and immigrants with origin in MENAPT countries (OR = 0.526) and in the other non-Western countries (OR = 0.532). In self-organised activities, large differences are found between citizens of Danish origin and immigrants and descendants with origin in MENAPT countries (OR_immigrants_ = 0.428 and OR_descendants_ = 0.508). Regarding participation in commercial centres, we only find immigrants from other non-Western countries (OR = 0.688) to participate less compared to citizens of Danish origin.

Across the three organisational forms, the analysis shows that descendants with origin in MENAPT countries and other non-Western countries are slightly more active than immigrants from the same countries of origin, in relation to the reference group. These differences are less pronounced among immigrants and descendants of Western origin. The findings in Table 3, which only includes immigrants and descendants, provide similar results regarding the differences between the origin groups to those in Table 2.

### Immigrant status

Table 2 shows that the higher the proportion of citizens within a local area that has a different ethnic origin than Danish, the lower is the general probability of participation in sports clubs. This applies both in relation to the proportion of immigrants and descendants of Western origin (OR = 0.979) as well as to the proportion of immigrants and descendants with origin in MENAPT countries and other non-Western countries (OR = 0.994) living in the local area. Similar results are found in both models in Table 3. Regarding participation in commercial centres, Table 2 shows that the higher the proportion of immigrants and descendants of Western origin living in the area, the higher the probability of participation (OR = 1.010). However, in both models in Table 3, which only includes immigrants and descendants, both variables regarding demography in local area are non-significant. Finally, in Table 2 the probability of participation in self-organised activities increases with the proportion of immigrants and descendants of Western origin living in the area (OR = 1.012) and decreases with the proportion of immigrants and descendants with origin in MENAPT countries and in other non-Western countries (OR = 0.993). In the model in Table 3 that includes both immigrants and descendants, the probability of participation in self-organised activities is found to decrease significantly with an increasing proportion of immigrants and descendants with origin in MENAPT countries and in other non-Western countries (OR = 0.994). In the model that includes only immigrants, both variables regarding demography in local area are non-significant.

In the model that includes only immigrants in Table 3, it is found that the probability of participation in sports clubs increases with the number of years the immigrants have been living in Denmark (OR = 1.003). The reverse is seen regarding participation in commercial centres (OR = 0.974). Further, the probability of participation in sports clubs (OR = 0.989) and in commercial centres (OR = 0.972) decreases with the age of the immigrant when arriving in Denmark.

### Sociodemographic and -economic characteristics

In Table 2, which includes the entire sample, almost all variables regarding sociodemographic and -economic characteristics are significantly correlated with physical activity participation in the three organisational forms. Only age is not significant in the models regarding physical activity participation in sports clubs and in self-organised activities, and further the variables regarding household composition and urbanisation level shows fewer significant correlations in relation to self-organised activities.

In Table 3, which only includes immigrants and descendants, we find the same correlation patterns for most sociodemographic and -economic variables as in the regression analysis of the entire population in Table 2. We focus our description on the results from Table 3, as our main focus is on the influence of sociodemographic and -economic variables on the physical activity participation of immigrants and descendants.

In terms of *gender*, both models in Table 3 shows that the probability for women – who are an immigrant or a descendant – to participate in sports clubs is lower compared to men (OR_immigrants and descendants_ = 0.692 and OR_immigrants_ = 0.756), while the opposite applies to participation in commercial centres (OR = 1.189_immigrants and descendants and_ OR = 1.328_immigrants_).

In terms of *age*, the model in Table 3 shows no correlation with immigrants and descendants’ participation in sports clubs and self-organised activities but a significant correlation with participation in commercial centres (OR = 0.972).

Regarding *education,* we find in both models in Table 3 that the probability of participation in sports clubs is lower for citizens with high school and vocational education (OR = 0.796_immigrants and descendants_ and OR = 0.756_immigrants_) compared to citizens with a higher education. For participation in commercial centres, the probability is lower for citizens whose highest education is primary school (OR = 0.845_immigrants and descendants_ and OR = 0.818_immigrants_). In self-organised activities, the probability is lower for both citizens with high school and vocational education and for citizens whose highest education is primary school (OR ≤ 0.661) compared to citizens with a higher education.

In terms of *employment*, Table 3 shows that the probability of participation in sports clubs is lower for employed people (OR = 0.786) and ‘others’ (OR = 0.713) compared to those who are studying. Further, in both models in Table 3 all employment groups are less likely to participate in self-organised activity compared to those who are studying (OR ≤ 0.805).

For the *income* variable*,* both models in Table 3 shows that citizens having a high income are more likely to participate in all organisational forms (OR_sports clubs_ ≥ 1.216, OR_commercial centres_ ≥ 1.556 and OR_self-organised_ ≥ 1.271), compared to those having a low income. For participation in commercial centres the same patterns are seen for those having a medium income (OR ≥ 1.183), compared to those having a low income.

In terms of *family composition,* Table 3 shows that immigrants living without a partner with children living at home have a lower probability for participation in sports clubs (OR = 0.778) compared to citizens living with a partner with children living at home. In commercial centres, the probability of participating is higher among all other groups than among citizens living with a partner with children living at home (OR ≥ 1.365) in both models. In self-organised activity, citizens living with a partner without children living at home (OR = 1.187_immigrants and descendants_ and OR = 1.234_immigrants_) have a higher probability of participation compared to citizens living with a partner with children living at home in both models, and for immigrants only this also applies to citizens living without a partner without children living at home (OR = 1.153).

Finally, regarding *urbanisation*, both models in Table 3 show that immigrants and descendants living in areas with 86–150 inhabitants per km^2^ are less likely to participate in sports clubs compared to citizens living in areas with 0–85 inhabitants per km^2^ (OR = 0.801_immigrants and descendants_ and OR = 0.778_immigrants_), and for immigrants only, this also applies to citizens living in areas 151–700 inhabitants per km^2^ (OR = 0.837). In commercial centres, there is a higher probability of participation among citizens living in areas with more than 150 inhabitants per km^2^ (OR ≥ 1.161) compared to citizens living in areas with 0–85 inhabitants per km^2^. For self-organised activities, the probability of participation is higher among immigrants living in areas with more than 700 inhabitants per km^2^ (OR = 1.174) compared to immigrants living in less urbanized areas.

The differences that do exist between Table 2 and Table 3 can be partly explained by the different sample sizes, while other differences likely reflect substantial differences in effects depending on whether citizens of Danish origin are included in the analysis. Jointly, the results indicate that sociodemographic and -economic characteristics to a higher extent influence the physical activity participation among citizens of Danish origin, compared to immigrants and descendants.

## Discussion

In the following, we will highlight some of the findings, and discuss them in relation to different societal levels. First, on a macro level, we will discuss the potential implications of our findings for Danish integration policy. Next, on a meso level, we discuss why all groups of immigrants and descendants are more active in commercial centres than in sports clubs. Finally, on a micro level, we reflect on the differences in physical activity participation between immigrants, descendants and the majority population.

### Danish integration policy

In relation to physical activity participation, our findings seem to justify the focus of Danish integration policy on the integration of immigrants and descendants with origin in MENAPT countries. However, our analysis underlines the need to also focus on integrating immigrants and descendants from other non-Western countries. This is because immigrants and descendants with a background from these countries are the least likely to be active in the three organisational forms. Our analysis shows how this can partly be explained by differences in sociodemographic and -economic factors, including that more immigrants and descendants have a short education, are not employed and have a low income. However, our analysis also reveals that the lower participation seems primarily to be related to ethnicity, i.e. country of origin.

Regarding integration in and through physical activity, the Danish integration policy almost exclusively centres on participation in sports clubs and other civil society organizations [28]. This approach could be debated based on our findings. On the one hand, the finding that all groups of immigrants and descendants use sports clubs significantly less than citizens of Danish origin could point to the need for an even stronger focus on sports clubs to reduce potential barriers for participation. On the other hand, the finding that there are smaller differences in the use of commercial centres could spur an interest in obtaining a better understanding of the potentials regarding integration in and through physical activity participation in commercial centres. However, it is an open question whether participation in a commercial centre to the same extent as participation in a sports club can foster a better understanding of Danish norms and values through social interactions and, thereby, lead to societal integration [31].

In sum, we encourage an integration policy with a particular focus on immigrants and descendants with origin in MENAPT countries and other non-Western countries, who are the least physically active in all organisational forms. Furthermore, we encourage policy makers not only to focus on integration in and through sports clubs, but also on other organisational forms for physical activity that can contribute to the integration of immigrants and descendants.

### Sports clubs versus commercial centres

As stated in previous section, and similar to previous studies [34, 47, 61], we found all groups of immigrants and descendants to be underrepresented in sports clubs compared to citizens with Danish origin. These differences are much smaller regarding participation in commercial centres. In the following we will elaborate on some of the potential explanations or barriers related to these differences.

A possible explanation for the differences can be sought in the work of Bourdieu [36] and Engström and colleagues [37]. They explain that participation in different types of physical activities relates to the individual’s habitus, which is affected by a person’s ethnic background. In Western countries, voluntary organizations, including sport clubs, have deep roots and are embedded in the social structures of society [62], which is not the case to the same extent in non-Western countries [63]. Immigrants and descendants with origin in MENAPT countries and other non-Western countries might therefore lack knowledge about organisational and social structures as well as the culture and norms present in sports clubs. Conversely, commercial centres and fitness activities are widespread in most countries, which can reduce the barriers for all groups of immigrants and descendants, as they are familiar with commercial centres from their country of origin. In fact, the results show that participation in sports clubs tends to increase with the number of years the immigrants have lived in Denmark. The reverse is seen regarding participation in commercial centres, and the probability of participation in sports clubs and in commercial centres also decreases with the age of the immigrant when arriving in Denmark. A possible explanation for this is that it takes time to build knowledge about relevant organisational forms.

The structure of sports clubs in Nordic and European countries, which is built around elements such as binding communities, volunteering, voluntary membership, and solidarity [64, 65], might also present a barrier for immigrants and descendants with origin in MENAPT-countries and other non-western countries. According to Rasmussen [63], ethnic minority groups are often attracted by open, informal, and unorganised activities, where they can be more spontaneously active. These preferences seem to better fit with commercial centres that are more individually organised, and customer driven [31]. As the commercial centres are more individually organised, one might also imagine that barriers related to language would be less common, which according to Rasmussen [63] is a central barrier related to participation among ethnic minorities in sports clubs.

Another potential explanation for the underrepresentation of immigrants and descendants in sports clubs could be related to the general overrepresentation of citizens of Danish origin in most sports clubs. This could lead to enhanced barriers for people with a different ethnic background, because individuals construct and preserve their identity by engaging in ethno-specific recreation [66]. The study from Koshoedo and colleagues [67] finds that some immigrants avoid participation in societal contexts they are unfamiliar with due to fear of either personal or institutional racial discrimination from the majority population.

In sum, it is our claim that the different structures, values, and norms present in the two organisational forms might explain the larger underrepresentation of all groups of immigrants and descendants in sports clubs relative to commercial centres observed in our study. A potential implication of this finding is that sports clubs might be inspired by commercial centres regarding how to become a more integrative setting.

### Differences between immigrants, descendants and the majority population

As stated in our analysis, we find that descendants are more likely to participate within all organisational forms compared to immigrants with the same origin (except for descendants with origin in other non-Western countries and their participation in self-organised activities). A likely explanation for this is that descendants have been part of the Danish society from childhood and have gained a larger understanding of Danish societal norms, values, and social interactions, for instance through school attendance. Also, they are likely to have gained knowledge about the various opportunities for being physically active at an early age, including in the organisational forms examined in this article [68, 69]. This suggests that physical activity participation at school is an important route for developing skills and familiarity with physical activity. It could therefore be beneficial for policy makers to prioritise fostering connections between schools and sports clubs, with a particular emphasis on individuals aged 15–16 years, ensuring their sustained engagement in physical activities beyond their school years.

The statistical analysis indicates that the lower participation in sports clubs and self-organised activities among immigrants and descendants compared to the Danish majority population cannot be explained by the fact that immigrants and descendants on average have a shorter education, lower income and are not employed to the same extent as citizens with a Danish background. The analysis does show that gender, age, place of residence (degree of urbanization), education, employment and income have an impact on immigrants and descendants' physical activity participation in the three forms of organization, but this also applies to the majority population. Either the differences in these correlations – between sociodemographic and -economic variables and participation in physical activity in different organizational forms – between the entire population (see Table 2) and immigrants and descendants (see Table 3) are small, or the sociodemographic and -economic characteristics to a lower extent influence the physical activity participation among immigrants and descendants compared to citizens of Danish origin.

The relatively higher participation of immigrants and descendants in commercial centres compared to their participation in sports clubs, even though participation in the former is significantly more expensive, is a further indication that sociodemographic and -economic characteristics has relatively little impact on the organizational context in which they are physically active. This calls into question the relevance of public programs that provide financial subsidies for the participation of immigrants and other vulnerable groups in sports clubs, where it is assumed that it is the costs that prevent many from becoming a member of a sports club.

In summary, it is our claim, first, that the differences in participation in physical activity in different organizational forms between the majority population and immigrants and descendants are primarily due to ethnicity and to a smaller extent to the greater sociodemographic and -economic inequality among immigrants and descendants. Second, it is our claim that the differences between immigrants and descendants with origin in MENAPT countries and other non-Western countries, regarding physical activity participation in sports clubs, commercial centres, and to some extend in self-organised activity is likely to be related to what can be described as an acculturation process. Because descendants are born in the host country, they are more likely to be familiar with society norms and values and to be familiar with different organisational forms for physical activity participation in the host country, which increases their likelihood of participation in adulthood.

### Limitations of the study

Our study has limitations that should be considered when interpreting the findings. First, the categorization of immigrants and descendants into relatively broad groups based on their respective countries of origin might overlook potential heterogeneities within these groups. However, limits to the extent to which immigrants and descendants can be further subdivided and still maintain enough respondents in the constructed groups to allow for valid statistical analyses formed the rationale for our choice.

Second, despite efforts to ensure the participation of immigrants and descendants in our study, including an additional targeted reminder in municipalities with relatively low response rates, they are still – as in most other survey studies – slightly underrepresented in our sample. This could potentially affect the generalisability of the comparisons made between groups according to ethnicity in this article because of variations in the response rates among these groups. However, although a drop-out analysis conducted as part of the ‘Moving Denmark’ project with 800 non-respondents showed that the non-respondents were less physically active than the survey respondents, most of the non-respondents were still physically active at least weekly. Hence, the potential bias introduced by differences in response rates among the compared groups is likely to be relatively minor.

## Conclusion

This article examined the role that ethnicity, immigrant status, and sociodemographic and -economic characteristics play for immigrants’ and descendants’ participation in sports clubs, commercial centres and self-organised activities in Denmark. Regarding ethnicity, we found that nearly all groups of immigrants and descendants across the different countries of origin participate significantly less in sports clubs and in self-organised activity compared to citizens of Danish origin. The same significant differences are not found regarding participation in commercial centres.

In relation to immigrant status, the analysis on immigrants only revealed that the longer immigrants have lived in Denmark, the greater is their likelihood of participating in sports clubs. A reverse pattern is found for participation in commercial centres. Additionally, for participation in sports clubs and commercial centres, the likelihood of participation decreases with the age of the immigrant upon arrival in Denmark. For the analysis on both immigrants and descendants, it is found that a higher proportion of citizens with another ethnic origin than Danish represented in the local area, the less likely it is that immigrants and descendants will participate in sports clubs. The proportion has none or the opposite influence on participation in commercial centres. For self-organised activities, immigrants and descendants are less likely to participate, when the proportion of immigrants and descendants with origin in MENAPT and other non-western countries in the area is high.

Finally, the analysis demonstrates that all sociodemographic and -economic variables exert some level of influence on participation among all groups of immigrants and descendants. Most important seems to be the level of education and income as it influences the level of participation across all organisational forms. Gender also plays a role, where women tend to be less active in sports clubs compared to men, while the opposite applies in commercial centres. Furthermore, for commercial centres, the family composition and for self-organised activities the employment status are also important variables influencing participation among immigrants and descendants.

Based on our findings, we encourage policymakers to have particular focus on immigrants and descendants with origin in MENAPT countries and other non-Western countries, who are the least physically active across all organisational forms, to focus on the benefits and the diversity of all organisational forms and to create initiatives that fosters integration in physical activities in and across all organisational forms.

## Data Availability

The datasets generated and/or analysed during the current study are not publicly available due to data protection regulations and the sensitive nature of some of the data collected in the survey study. For any queries regarding the data, please contact the corresponding author Eva Berthelsen Schmidt, at evaschmidt@health.sdu.dk.

## Abbreviations

SES: Socioeconomic status
MENAPT countries: Middle East and North African countries and Turkey
